# Correction to “Interoceptive Information of Physical Vigor Through Circulating Insulin‐Like Growth Factor 1”

**DOI:** 10.1096/fba.2026-00036

**Published:** 2026-02-11

J. A. Zegarra‐Valdivia, M. Z. Khan, J. Fernandes, et al., “Interoceptive Information of Physical Vigor Through Circulating Insulin‐Like Growth Factor 1,” *FASEB BioAdvances* 8, no. 1 (2026): e70084, https://doi.org/10.1096/fba.2025‐00226.

In the original paper, the name of one of the authors was incorrect. It was incorrectly stated as “M. Zahid Kahn.” The author's correct name is “M. Zahid Khan.”

Additionally, in the original paper, Supplementary Figures: S1B, S2B, S2D, S3, S4B, S4D, S4E, S5A, S5B, S5C, S5E, and S6D contained blank panels. The correct graphical data that were peer reviewed have been returned to the supplementary figures.

We apologize for these errors.

